# Ansamitocin P3 Depolymerizes Microtubules and Induces Apoptosis by Binding to Tubulin at the Vinblastine Site

**DOI:** 10.1371/journal.pone.0075182

**Published:** 2013-10-04

**Authors:** Jubina B. Venghateri, Tilak Kumar Gupta, Paul J. Verma, Ambarish Kunwar, Dulal Panda

**Affiliations:** 1 IITB-Monash Research Academy, Indian Institute of Technology Bombay, Mumbai, India; 2 Department of Biosciences and Bioengineering, Indian Institute of Technology Bombay, Powai, Mumbai, India; 3 Centre for Reproduction and Development, Monash Institute of Medical Research, Monash University, Clayton, Victoria, Australia; University of Alberta, Canada

## Abstract

Maytansinoid conjugates are currently under different phases of clinical trials and have been showing promising activity for various types of cancers. In this study, we have elucidated the mechanism of action of ansamitocin P3, a structural analogue of maytansine for its anticancer activity. Ansamitocin P3 potently inhibited the proliferation of MCF-7, HeLa, EMT-6/AR1 and MDA-MB-231 cells in culture with a half-maximal inhibitory concentration of 20±3, 50±0.5, 140±17, and 150±1.1 pM, respectively. Ansamitocin P3 strongly depolymerized both interphase and mitotic microtubules and perturbed chromosome segregation at its proliferation inhibitory concentration range. Treatment of ansamitocin P3 activated spindle checkpoint surveillance proteins, Mad2 and BubR1 and blocked the cells in mitotic phase of the cell cycle. Subsequently, cells underwent apoptosis via p53 mediated apoptotic pathway. Further, ansamitocin P3 was found to bind to purified tubulin *in vitro* with a dissociation constant (K_d_) of 1.3±0.7 µM. The binding of ansamitocin P3 induced conformational changes in tubulin. A docking analysis suggested that ansamitocin P3 may bind partially to vinblastine binding site on tubulin in two different positions. The analysis indicated that the binding of ansamitocin P3 to tubulin is stabilized by hydrogen bonds. In addition, weak interactions such as halogen-oxygen interactions may also contribute to the binding of ansamitocin P3 to tubulin. The study provided a significant insight in understanding the antiproliferative mechanism of action of ansamitocin P3.

## Introduction

**Figure 1:**
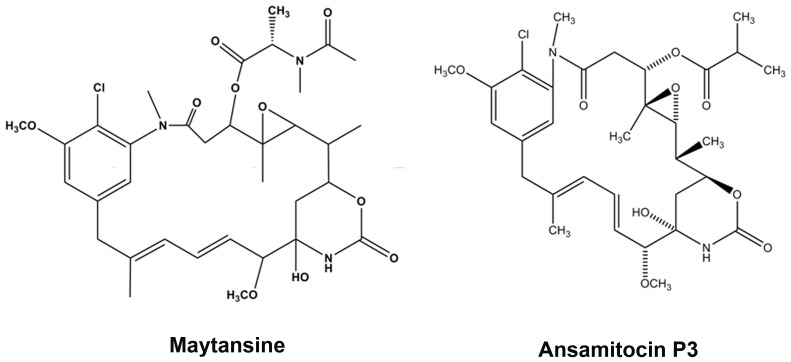
Structures of maytansine and ansamitocin P3 are shown.

Potent cytotoxic agents like maytansine (Fig.1) are entering clinical trials with significant improvements mainly by conjugation with tumor specific antibodies [1], [2]. Recent clinical trials of maytansine conjugated antibodies namely, trastuzumab emtansine (under Phase III), SAR3419 and BT062 (under Phase II), and several others such as BAY 94–9343, BIIB015, IMGN529, lorvotuzumab mertansine, SAR566658, IMGN529 under Phase I clinical trial have strengthened the hope of targeting tumor cells with highly potent cytotoxic agents that were previously withdrawn from cancer chemotherapeutic regimen [1]. Maytansinoids are potent microtubule targeting cytotoxic agents and had shown promising activity in B-16 melanocarcinoma murine solid tumors in addition to anti leukemic activity against P388 murine lymphocytic leukemia [3]. Maytansinoids are known to exhibit 100 to 1000 times more cytotoxicity than several of the known anticancer agents [2], [4]. However, severe side effects involving neuronal and gastrointestinal toxicity along with narrow therapeutic index displayed by maytansinoids in clinical trials led to their fall in cancer therapy [3]. Current progress in antibody drug conjugate mediated anti-cancer therapy has revived the interest in maytansine. Accordingly, this has opened the door for other structural analogues of maytansine that may provide abundant opportunities for antibody drug conjugate study and thereby offer numerous options for cancer chemotherapy. The maytansinoid family comprises of several structural analogues such as maytansinol, maytansine, maytansinol 3-propionate, ansamitocin P3, ansamitocin P4 [5]. Maytansine has been known to exert its antimitotic activity by inhibiting the assembly of microtubules and blocking the cells at mitosis [6]–[8]. Recent studies have shown that antibody maytansinoid conjugates block the cells at mitosis by suppressing the microtubule dynamics similar to the results obtained involving the unconjugated maytansine [9], [10].

Ansamitocin P3 (Fig. 1), a structural analogue of maytansine, was isolated from *Nocardia* [11], [12]. Structurally maytansine and ansamitocin P3 (Fig. 1) are polyketide macrolactams differing in the acyl groups at the C3 position [12]–[16]. Ansamitocin P3 has been known to depolymerize microtubules and bind to tubulin in a competitive manner with vinblastine and rhizoxin suggesting that it partially overlaps the vinblastine binding site [5], [16]–[18] **.** Current promising trials of maytansinoid drug conjugates have urged us to probe the mechanism of action of ansamitocin P3. In the present study, we have elucidated the antiproliferative activity of ansamitocin P3. Treatment of MCF-7 cells with ansamitocin P3 resulted in severe disruption of interphase and mitotic microtubules. The affected cells were blocked in mitosis and accumulated p53 and its downstream partner p21 in the nucleus, which activated apoptotic cell death in these cells. In addition, molecular docking analysis indicated a putative binding site of ansamitocin P3 on tubulin and elucidated the nature of interaction involved in the binding of ansamitocin P3 to tubulin.

## Materials and Methods

### Materials

Ansamitocin P3, sulforhodamine B (SRB), Hoechst 33258, 5,5′-dithiobis-2-nitrobenzoic acid (DTNB), mouse monoclonal anti-α tubulin IgG, alkaline phosphatase-conjugated anti-mouse and anti-rabbit IgG were procured from Sigma (St Louis, MO, USA). Fetal bovine serum was procured from Biowest (Nuaille, France). Bovine serum albumin (BSA), Dulbecco’s phosphate buffered saline (DPBS) were purchased from HiMedia, Mumbai, (India). For apoptosis detection, FITC Annexin apoptosis detection kit was purchased from BD Biosciences (San Jose, CA, USA). Mouse anti-p53 IgG, mouse anti-p21 IgG, rabbit polyclonal anti-PARP IgG and rabbit anti-phosphohistone-H3 (serine 10) IgG antibodies were purchased from Santa Cruz Biotechnology (CA, USA). Mouse anti-BubR1 antibody was purchased from BD Biosciences (San Jose, CA, USA). Rabbit polyclonal anti-Mad2 IgG antibody was obtained from Bethyl laboratories (Montgomery, USA). Rabbit polyclonal anti-α tubulin IgG was purchased from Abcam (Cambridge, MA, USA). Alexa fluor 568 conjugated anti-mouse IgG was purchased from Molecular Probes, Invitrogen (Eugene, USA). All the other reagents utilized for the experiments were of analytical grade.

### Cell Culture

Human breast adenocarcinoma (MCF-7) and human cervical carcinoma (HeLa) cells were grown in minimal essential media (HiMedia, Mumbai, India) supplemented with 2.2 g/L of sodium bicarbonate (HiMedia, Mumbai, India) and 10% (v/v) fetal bovine serum (FBS) [19]. In addition, the medium was supplemented with 1% antibiotic–antimycotic solution (HiMedia, Mumbai, India) comprising of streptomycin, amphotericin B, and penicillin. MDA-MB-231 cells were cultured in Leibovitz’s L-15 medium (Hi Media, India) as described previously [19]. All cell lines were cultured at 37°C in a humidified chamber (Sanyo, Tokyo, Japan) with 5% CO_2_. Multi-drug resistant mouse mammary tumor (EMT-6/AR1) cell line was cultured as described previously [19].

### Effects of Ansamitocin P3 on Cell Proliferation

MCF-7, EMT-6/AR1, HeLa and MDA-MB-231 cells were seeded in 96 well plates. Subsequently, cells were incubated with vehicle (0.1% DMSO) or different concentrations (1–1000 pM) of ansamitocin P3 for 48 h in MCF-7 cells and 24 h for EMT-6/AR1, HeLa and MDA-MB-231 cells, respectively. The half maximal inhibitory concentration of cell proliferation (IC_50_) for ansamitocin P3 was determined by sulforhodamine B assay [20]. Four independent experiments were carried out in MCF-7 cells and three independent sets of experiments were performed in EMT-6/AR1, HeLa and MDA-MB-231 cells.

### Effects of Ansamitocin P3 on Cell Cycle Progression in MCF-7 Cells

MCF-7 cells were treated with either vehicle or different concentrations (20–100 pM) of ansamitocin P3 for 24 h. Cells were pelleted, fixed in 70% ethanol in PBS and subsequently, the fixed cells were incubated with 50 µg/mL of propidium iodide (PI) and 8 µg/mL of RNAse for 2 h at 4°C [21]. The flow cytometry analysis of the cells was carried out by BD FACS Aria special order system (Becton Dickinson, San Jose, CA, USA). The data obtained were then fit using Modfit LT version 3.2 (Verity Software House, ME, USA).

### Mitotic Index Calculation

MCF-7 cells (1×10^5^ cells/mL) were seeded on glass coverslips for 24 h. Cells were then incubated with either vehicle (0.1% DMSO) or different concentrations (20–100 pM) of ansamitocin P3 for 24 h. The cells were collected by centrifugation (1200 g at 30°C) using a Labofuge 400R cytospin (Heraeus, Hanau, Germany) for 10 min and fixed with 3.7% formaldehyde for 20 min at 37°C. Finally, the cells were permeabilized with chilled methanol and stained with Hoechst 33258 (1µg/mL) [22]. The number of cells in mitosis and interphase were counted for both the vehicle control and ansamitocin P3 treated cells using the Eclipse TE2000-U microscope (Nikon, Tokyo, Japan). The experiment was performed three times.

### Immunofluorescence Microscopy

MCF-7 cells (0.5×10^5^ cells/mL) were seeded on glass coverslips in a 24 well tissue culture plate for 24 h [19]. Subsequently, cells were incubated without or with different concentrations of ansamitocin P3 for an additional 24 h. The cells were fixed with 3.7% formaldehyde for 20 min at 37°C and washed with PBS twice. Cells were then permeabilized with chilled methanol at −20°C for 15 min and washed with PBS twice. To prevent non-specific binding of antibodies, coverslips were incubated with 2% BSA in PBS for 1 h at 37°C. Immunostaining for tubulin, p53, p21, phosphohistone H-3 (Ser 10), Mad2 and BubR1 were performed as described recently [19]. Images were collected using a Nikon Eclipse TE2000-U microscope and analyzed using Image-Pro Plus software (Media Cybernetics, MD, USA).

### Western Blot Analysis

MCF-7 cells were incubated in the absence or presence of 100 and 150 pM ansamitocin P3 and 25 nM vinblastine, respectively for 24 h. Whole cell lysates were prepared and 100 µg of samples were ran on SDS poly acrylamide gel electrophoresis. Subsequently, blotted on a PVDF membrane and Western blot was performed [22]. Briefly, the membrane was blocked using 5% skim milk in TBST buffer and then, incubated with primary antibodies rabbit polyclonal anti-PARP IgG, mouse monoclonal β- actin IgG (1∶1000), mouse monoclonal anti-BubR1 and recognized using an alkaline phosphatase conjugated anti-mouse IgG (1∶1000) or anti rabbit (1∶1000) antibody.

### Apoptosis Assay Using Annexin V-PI Staining

MCF-7 cells were treated with either vehicle or different concentrations of ansamitocin P3 for 24 h. The Annexin V-propidium iodide staining was performed using Annexin V-FITC propidium iodide apoptosis detection kit (BD Bisociences, USA) [23] and the flow cytometry analysis of the cells was carried out.

### Tubulin Isolation

Tubulin was isolated from goat brain by repeated cycles of polymerization and depolymerization using 1 M glutamate [24], [25]. The protein was stored at −80°C. The concentration of tubulin was estimated by Bradford method [26] using bovine serum albumin as standard.

### Dissociation Constant of Ansamitocin P3 to Tubulin

Tubulin (2 µM) was incubated without or with different concentrations (0.5–15 µM) of ansamitocin P3 for 10 min at 25°C. Emission spectra (310–370 nm) were collected using a JASCO FP-6500 spectrofluorometer by exciting the samples at 295 nm. A 0.3 cm path length cuvette was used. Ansamitocin P3 has no significant absorbance at either excitation or emission wavelength. Therefore, inner filter effect correction was not required. Respective blank values were subtracted from all the measurements. The dissociation constant (K_d_) was calculated by fitting the fluorescence data in the equation
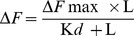
where L is the concentration of ansamitocin P3, ΔF is the change in the fluorescence intensity of the tubulin when bound to ansamitocin P3; ΔF_max_ is the maximum change in the fluorescence intensity of tubulin when it is fully bound with ansamitocin P3. The K_d_ was calculated using Graph Pad Prism 5 software (Graph Pad Software, CA, USA). The experiment was performed 5 times.

### Titration of Sulfhydryl Groups

Tubulin (5 µM) was incubated without or with 3 and 5 µM ansamitocin P3 on ice for 20 min, and then 300 µM DTNB was added [25], [27]. The absorbance was monitored for 45 min in a spectrophotometer at 25°C. The number of cysteine residues reacted after 45 min was determined by using a molar extinction coefficient of 12 000 M^−1^ cm^−1^ for TNB^−^ at 412 nm [25], [27] **.** The linear rate of sulfhydryl modifications was obtained by plotting 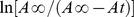 versus time where A_∞_ *and* A_t_ are the absorbance of TNB^−^ at saturation state and at different times.

### Molecular Docking

Molecular docking was performed using Autodock 4.2 [28], to identify the putative binding site of ansamitocin P3 and maytansine on tubulin dimer as described recently [29]. PRODRG server [30] was used to generate an energy minimized three dimensional atomic-coordinate of ansamitocin P3 and maytansine. We used the structure of α-β tubulin dimers (PDB ID 1Z2B) bound with RB3 stathmin-like domain with two molecules of bound DAMA-colchicine and one molecule of bound vinblastine for protein coordinates [31]. Only the minus-end α subunit (C chain) and plus-end β-subunit (B chain), both of which interact with vinblastine was utilised as a template for the docking studies. However, A and D subunits along with other ligands including stathmin like domain and colchicine were removed from the protein structure prior to perform the docking simulation. Prior to the start of docking, essential hydrogens was added to tubulin using UCSF Chimera interface [32]. Initially, blind docking for vinblastine, ansamitocin P3 and maytansine was performed on tubulin. For blind docking [33], [34], the entire tubulin dimer was enclosed in a grid box of 126×126×126 grid points with a grid spacing of 0.80 Å, keeping tubulin rigid and drug as a flexible molecule. The Lamarckian genetic algorithm (LGA) was employed with the default parameters; g_eval was set to 2,500,000 (Medium). Five independent docking jobs were conducted, each of 100 runs so that 500 output conformations were obtained. For each docking job, a ligand was placed at random locations. It was found that all ligands were going at the interface. Therefore, local docking analysis was performed at the interface only. For local docking, the grid box was made in such a way that it covered entire interface of α-β tubulin dimer along with maximum possible search space around interface. For this purpose, a grid box of 126×126×126 grid point with grid spacing 0.375 Å was constructed at the interface of tubulin dimer. The Lamarckian genetic algorithm (LGA) was employed with the default parameters; g_eval was set to 2,500,000 (Medium). 50 independent flexible ligand docking jobs each of 100 LGA runs were conducted for all three drugs, which produced 5000 output conformation. Autodock 4.2 was used to perform clustering of binding conformations based on similarities in binding modes and affinities. The optimized conformations represent possible binding modes of the drug binding on tubulin [28], [35].The resulted 5000 output conformations were clustered using an all-atom RMSD cut-off of 5 Å and the clusters having more than 40 conformations (cut-off number) were analyzed further. The clusters were compared on the basis of the cluster size, solvent accessible surface area and binding energy calculated by AutoDock 4.2 scoring function. Molecular graphics and analysis were performed using a Chimera package [32], which has been designed by the Resource for Biocomputing, Visualization, and Informatics at the University of California San Francisco (supported by NIGMS P41-GM103311).

## Results

### Ansamitocin P3 Inhibited the Proliferation of Several Types of Cancer Cells in Culture

**Figure 2:**
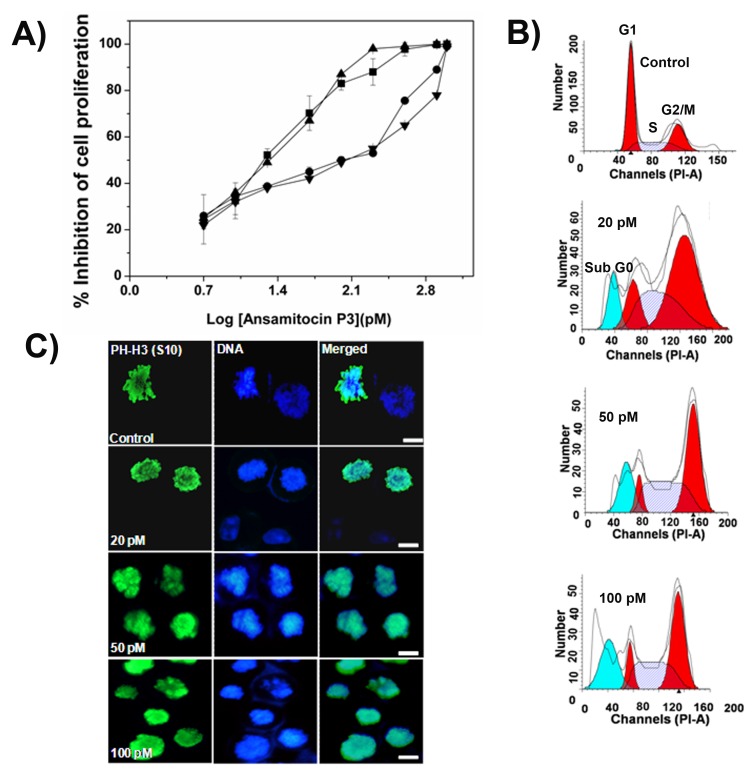
Effects of ansamitocin P3 on cell proliferation and cell cycle progression. (A) Inhibition of MCF-7 (▪), HeLa (▴), EMT-6/AR1 (•) and MDA-MB-231(▾) cell proliferation by ansamitocin P3. (B) Exposure of MCF-7 cells to ansamitocin P3 for 24 h increased the percentage of cells in G2/M phase of the cell cycle. (C) Ansamitocin P3 treatment increased the percentage of phosphohistone [PH-H3 (S10)] stained cells. Scale bar is 10 µm. MCF-7 cells were incubated with vehicle (control) and different concentrations (20, 50 and 100 pM) of ansamitocin P3 for 24 h.

Ansamitocin P3 (Fig. 1) inhibited the proliferation of MCF-7, HeLa, EMT-6/AR1 and MDA-MB-231 cells in culture in a concentration dependent manner (Fig. 2A). The half-maximal proliferation inhibitory concentrations (IC_50_) of ansamitocin P3 in MCF-7, HeLa, EMT-6/AR1 and MDA-MB-231 cells were determined to be 20±3, 50±0.6, 140±17, and 150±1.1 pM, respectively (Fig. 2A). Flow cytometric analysis of PI-stained cells suggested that ansamitocin P3 inhibited the cell cycle progression of MCF-7 cells in G2/M phase (Fig. 2B). For example, 26, 50 and 70% of the cells were found to be in G2/M phase in the absence and presence of 50 and 100 pM ansamitocin P3, respectively. The mitotic index [(number of cells in mitosis/total number of cells) ×100] was determined to be 3±0.5, 23±3, 33±0.8, and 44±4 (p<0.001) in the absence and presence of 20, 50 and 100 pM ansamitocin P3, respectively, suggesting that ansamitocin P3 treatment induced mitotic block in MCF-7 cells. Further, the number of cells stained with phosphohistone-H3 (serine 10) was found to be 3±0.5, 14±1.2, 21±0.5 and 29±0.6% (p<0.006), in the absence and presence of 20, 50 and 100 pM ansamitocin P3, respectively (Figure 2C). The results also suggested that ansamitocin P3 treatment blocked cells in mitosis (Figure 2C).

### Ansamitocin P3 Perturbed the Microtubule Organization in MCF-7 Cells

**Figure 3:**
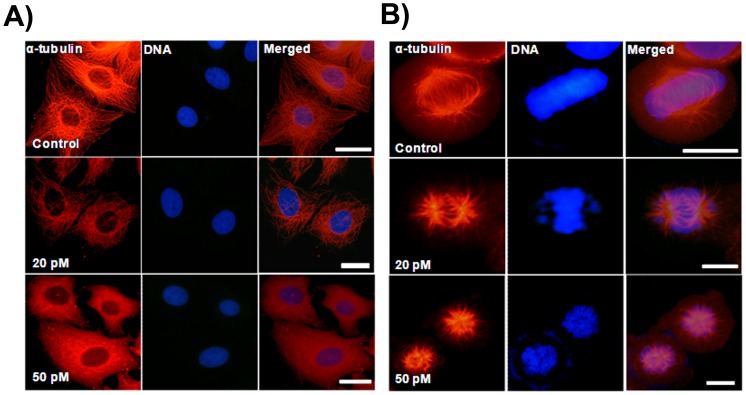
Ansamitocin P3 caused depolymerization of microtubules in MCF-7 cells. MCF-7 cells were incubated in the absence or presence of different concentrations of ansamitocin P3 for 24 h. Interphase (A) and mitotic (B) microtubules are shown. Scale bar is 10 µm.

Ansamitocin P3 disrupted interphase microtubule organization of MCF-7 cells (Fig. 3A). The depolymerizing effects increased with increasing concentration of ansamitocin P3. Ansamitocin P3 (20 pM) depolymerized interphase microtubules to some extent (Fig. 3A). In the presence of 50 pM ansamitocin P3, strong depolymerization of interphase microtubules was observed (Fig. 3A). Ansamitocin P3 produced much stronger depolymerizing effects on mitotic microtubules than the interphase microtubules (Fig. 3B). For example, microtubules in mitotic cells were strongly depolymerized in the presence of 50 pM ansamitocin P3 and spindles were severely distorted in these cells (Fig. 3B). In vehicle treated mitotic cells, the chromosomes were condensed and organized at the metaphase plate (Fig. 3B). In the presence of 20 pM ansamitocin P3, a few chromosomes were not aligned properly at the metaphase plate while 50 pM ansamitocin P3 treatment severely distorted chromosome organization in the mitotic cells (Fig. 3B).

### Ansamitocin P3 Activated Spindle Assembly Checkpoint Proteins Mad2 and BubR1

**Figure 4:**
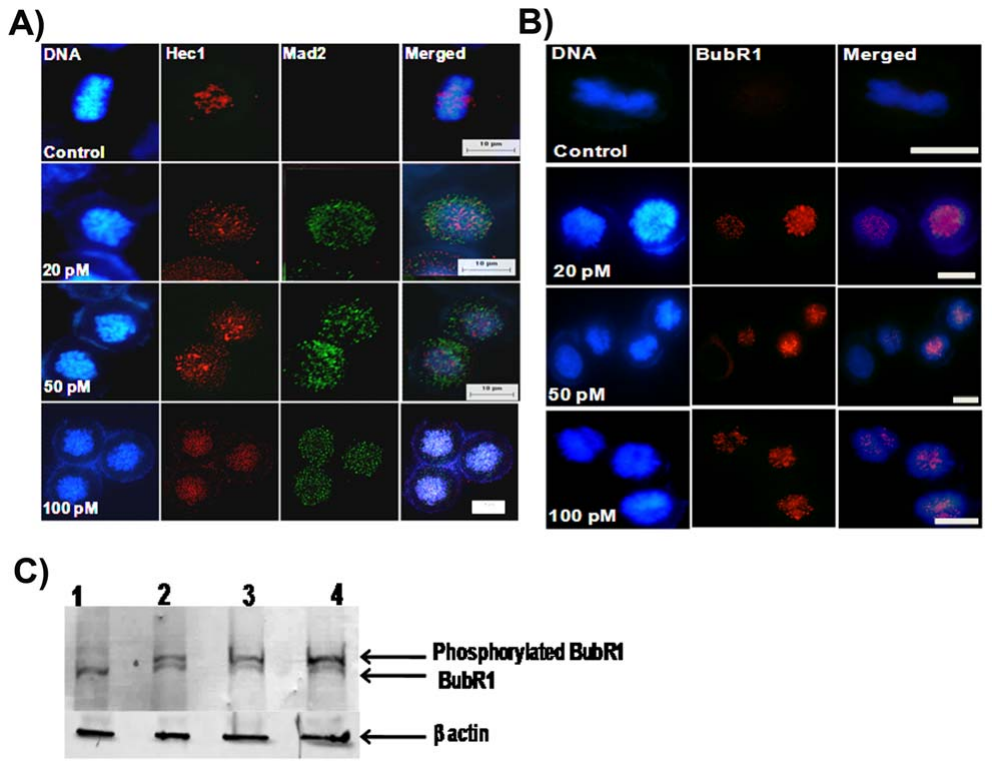
Ansamitocin P3 treatment activated mitotic checkpoint proteins. Ansamitocin P3 caused the accumulation of Mad 2 (A) and BubR1 (B). MCF-7 cells were incubated in the absence or presence of different concentrations (20, 50 and 100 pM) of ansamitocin P3 for 24 h. Cells were also stained with Hec1 to show the position of the kinetochore. Scale bar is 10 µm. (C) Ansamitocin P3 treatment increased the phosphorylation of BubR1 in MCF-7 cells. Lanes 1, 2, 3 and 4 represent control, 100 and 150 pM of ansamitocin P3, and 25 nM of vinblastine, respectively. β-actin was used as a loading control.

Mad2 and BubR1 are known to sense the microtubule kinetochore attachment or tension across the kinetochores [36]. In control mitotic cells, Mad2 and BubR1 were not found to be accumulated at the kinetochores (Fig. 4A and 4B). However, in ansamitocin P3 treated cells Mad2 was localized at the kinetochores (Fig. 4A). Similarly, BubR1 was found to be accumulated in the nucleus of ansamitocin P3 treated MCF-7 cells (Fig. 4B). In addition, ansamitocin P3 was found to increase the phosphorylation level of BubR1 in MCF-7 cells indicating that it activated BubR1 (Fig. 4C). The results together indicated that ansamitocin P3 activated the mitotic checkpoint in MCF-7 cells.

### Ansamitocin P3 Induced Apoptosis in MCF-7 Cells

**Figure 5:**
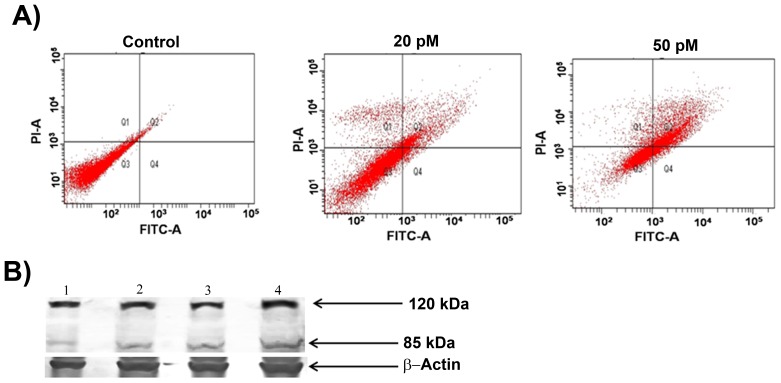
Ansamitocin P3 induced apoptosis of MCF-7 cells. (A) The apoptosis in MCF-7 cells was estimated by Annexin V and PI staining followed by flow cytometry analysis. Quadrants represent dead (Q1), late apoptotic (Q2), live (Q3) and (Q4) early apoptotic cells. (B) Ansamitocin P3 treatment caused cleavage of PARP. Lanes 1, 2, 3 and 4 represent control, 100 and 150 pM ansamitocin P3 and 25 nM vinblastine treatment of MCF-7 cells respectively. β-actin was used as loading control.

**Figure 6:**
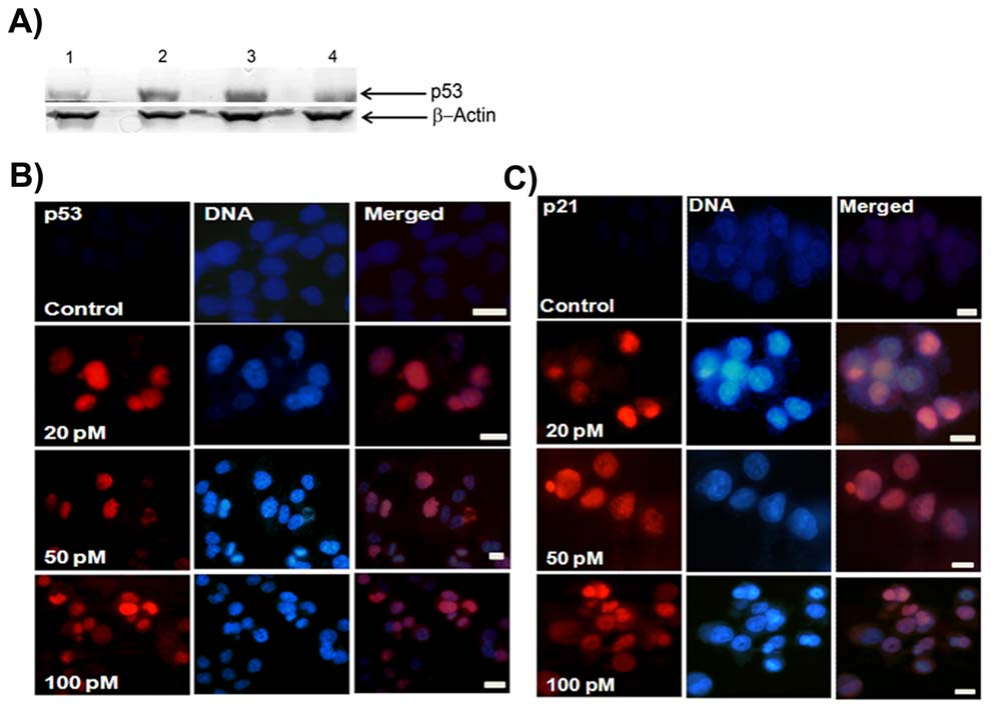
Ansamitocin P3 treatment activated p53. (A) Ansamitocin P3 treatment caused an increase in the expression level of p53 in MCF-7 cells. Lanes 1, 2, 3, and 4 represent control, 100 and 150 pM of ansamitocin P3 and 25 nM vinblastine treated cells, respectively. β-actin was used as loading control. Ansamitocin P3 treatment increased the nuclear accumulation of p53 (B) and p21 (C). MCF-7 cells were incubated without or with different concentrations (20, 50 and 100 pM) of ansamitocin P3 for 24 h. Scale bar is 10 µm.

Ansamitocin P3 treatment caused apoptosis in MCF-7 cells as evident from the Annexin V and propidium iodide staining of the treated cells (Fig. 5 and Figure S1). For example, the percentage of dead cells increased from 3% in control to 50% in case of 50 pM treated cells (Table 1). The cleavage of Poly (ADP-ribose) polymerase (PARP) is a known marker for apoptosis [23], [37]. In vehicle-treated MCF-7 cells, a single band of PARP was detected whereas ansamitocin P3 treated cells displayed two bands of PARP protein indicating the cleavage of PARP (Fig. 5B). The result suggested that ansamitocin P3 treatment induced apoptosis in MCF-7 cells. Since ansamitocin P3 treatment induced apoptosis in MCF-7, we examined whether it activated p53 in these cells. Western blot analysis suggested that the expression level of p53 increased in ansamitocin P3 treated cells as compared to the vehicle treated cells (Fig. 6A). Therefore, we examined the localization of p53 in ansamitocin P3 treated cells. The treatment of MCF-7 cells with ansamitocin P3 increased the nuclear accumulation of p53 (Fig. 6B). To examine whether the downstream targets of p53 are activated upon p53 nuclear accumulation, the localization of p21 in ansamitocin P3 treated MCF-7 cells was analyzed (Fig. 6C). p21 was found to be accumulated in nucleus of the ansamitocin P3 treated MCF-7 cells while it was absent in the vehicle treated cells indicating the activation of p53 dependent genes in ansamitocin P3 treated cells (Fig. 6C).

**Table 1:** Ansamitocin P3 induced apoptosis in MCF- cells.

Sample	% Live Cells	% Dead Cells
**Control**	97	3
**20 pM**	75	25
**50 pM**	47	53

### Dissociation Constant of Ansamitocin P3 to Tubulin

**Figure 7:**
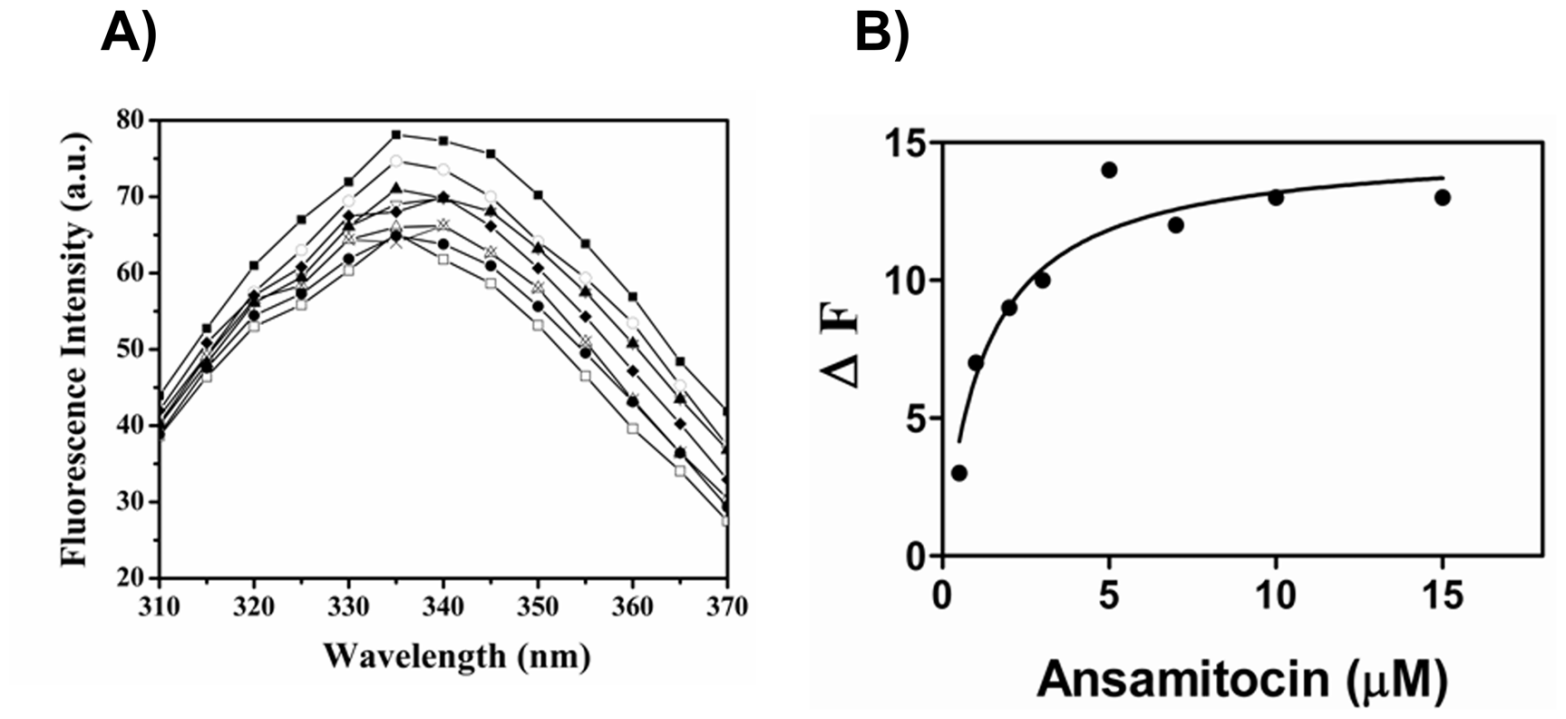
Ansamitocin P3 quenched tryptophan fluorescence of tubulin. (A) Effects of ansamitocin P3 on the intrinsic tryptophan fluorescence of tubulin are shown. Tubulin was incubated in 25 mM PIPES buffer (pH 6.8) without (**▪**) and with different concentrations 0.5 (○), 1 (▴), 2 (∇), 3 (♦), 5 (**×**), 7 (Δ), 10 (□) and 15 (•) µM of ansamitocin P3, respectively. (B) The dissociation constant was determined from the fluorescence change data using Graph Pad Prism 5 software The K_d_ was average of 5 data sets.

Intrinsic tryptophan fluorescence of tubulin decreased in a concentration dependent manner upon incubation with increasing concentrations of ansamitocin P3 (Fig. 7A). A dissociation constant (K_d_) for the binding of ansamitocin P3 to tubulin was estimated to be 1.3±0.7 µM by fitting the fluorescence change data in a binding equation (Fig. 7B).

### Ansamitocin P3 Modified the Cysteine Residues of Tubulin

**Figure 8:**
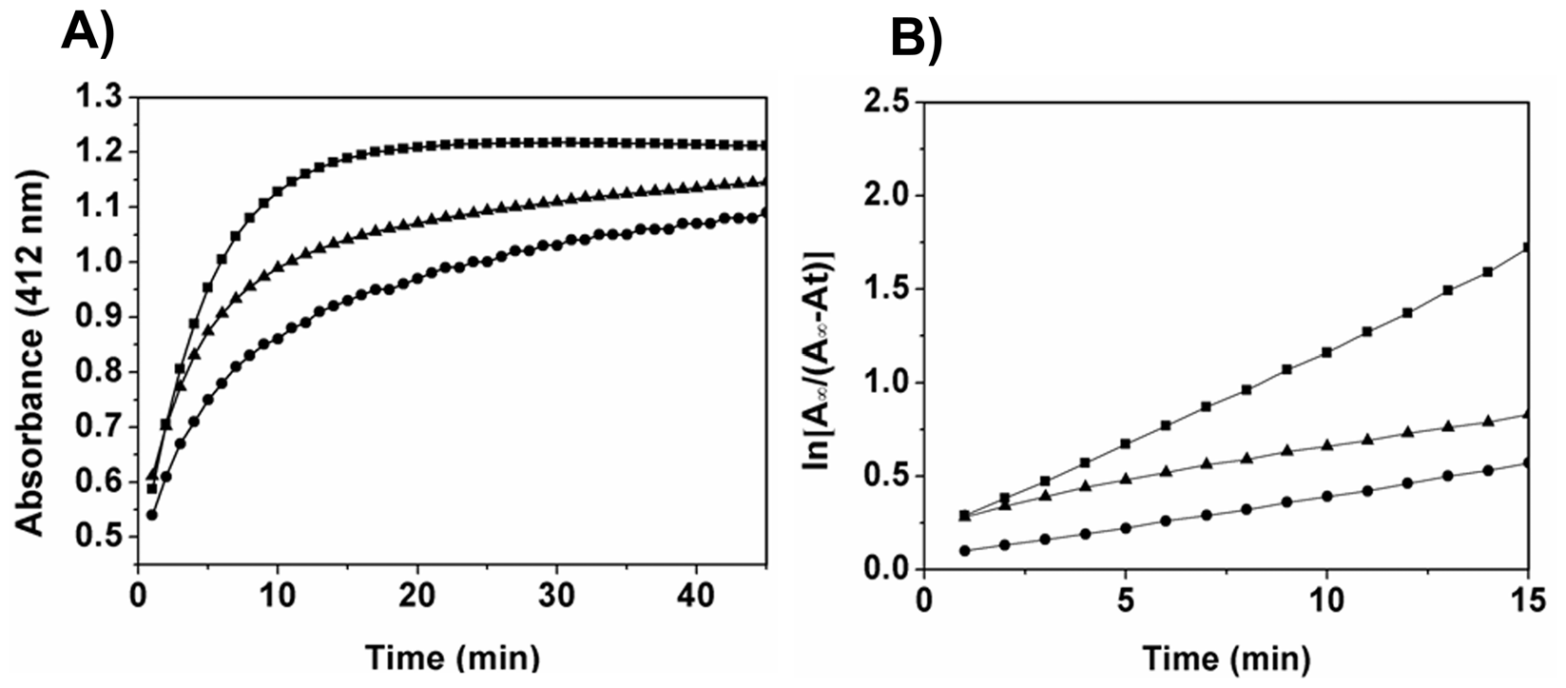
Ansamitocin P3 suppressed chemical modification of cysteine residues of tubulin by DTNB. (A) Kinetics of modification of tubulin by DTNB in the absence (**▪**) and presence of 3 µM (▴), 5 µM (•) of ansamitocin P3 are shown. The absorbance was monitored at 412 nm. (B) The pseudo first order plots of sulfhydryl modifications in the absence (**▪**) and presence of 3 µM (▴) and 5 µM (•) ansamitocin P3 are shown. One of three experiments is shown.

The binding of colchicine and vinblastine to tubulin is known to reduce the number of accessible cysteine residues for chemical modification [38]. Fig. 8A shows the reaction kinetics of cysteine modification of tubulin with DTNB, in the absence and presence of 3 and 5 µM of ansamitocin P3. The average number of accessible cysteine residues per tubulin dimer was reduced from 12±0.02 to 11±0.01 and 9±0.01 upon treatment with 3 and 5 µM of ansamitocin P3, respectively (p<0.001). In addition, ansamitocin P3 was also found to reduce the initial rate of cysteine modification by DTNB indicating that the binding of the ligand induces conformational change in tubulin (Fig. 8B).

### Ansamitocin P3 and Maytansine Bind Partially to Vinblastine Binding Site of Tubulin

**Figure 9:**
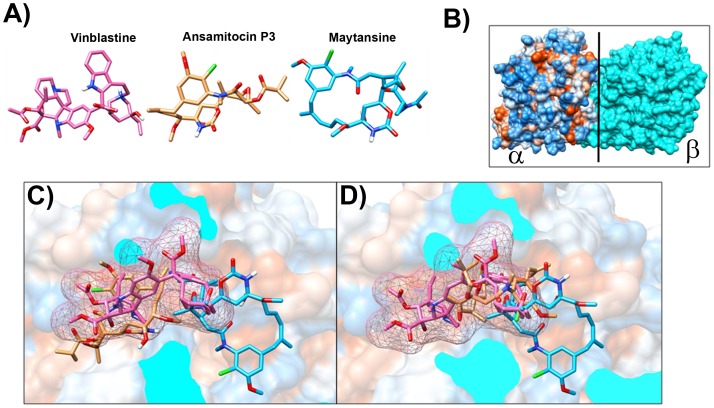
Ansamitocin and maytansine bind at the vinblastine binding pocket in tubulin dimer. (A) Structures of vinblastine (Pink), ansamitocin P3 (brown) and maytansine (blue) are shown. (B) Surface view of α-β tubulin dimer, α chain is shown in hydrophobic surface while β chain is shown in cyan color. Vertical black line shows the axis where the β chain is sliced out to show the cross-sectional views in (C) and (D). (C) and (D) show the cross-sectional view of the vinblastine binding site. The two binding orientations, position A and position B, of ansamitocin P3 (brown sticks) are shown in (C) and (D), respectively. Vinblastine is depicted with pink sticks and mesh surface and maytansine is shown in blue sticks. Ansamitocin P3, in position A, is partially overlapping with vinblastine binding pocket while ansamitocin P3, in position B, is partially overlapping with vinblastine binding pocket and partially with maytansine binding pocket. Interestingly, maytansine binding site also partially overlaps with vinblastine binding pocket.

Ansamitocin P3 was shown to inhibit the binding of vinblastine to tubulin with an inhibition constant (K_i_) of 1.5×10^−8^ M [18]. The binding sites of maytansinoids were suggested to partially overlap with the vinblastine binding site on tubulin [39], [40]. Therefore, docking with vinblastine on tubulin dimer as a control was performed first. The docked conformation was compared with X-ray crystallographically determined structure of vinblastine. The binding energy of the minimum energy docked conformations (Fig. 9) is −11.34 kcal/mole (Table 2). The root mean square deviation (RMSD) between the “predicted” and crystallographically determined binding modes of vinblastine was 0.8 Å. RMSD of less than 2 Å indicates an acceptable docking analysis [28], [41]. Docking of ansamitocin P3 on tubulin was performed using the same procedure as for the control (vinblastine). The analysis suggested that ansamitocin P3 might bind to tubulin at vinblastine binding site in two positions (position A and position B) (Fig. 9). The estimated binding energies for position A and B were −10.27 and −10.18 kcal/mole respectively, indicating that ansamitocin P3 may bind to tubulin in both conformations as the estimated energy difference between these two conformations is not too large. Since both the positions had comparable energy (−10.27 and −10.18 kcal/mole), we also calculated solvent accessible surface area by using NACCESS [42] to find out how well ansamitocin P3 was buried in vinblastine binding pocket in both the positions. Values of solvent accessible surface area for ansamitocin P3 after binding to tubulin dimer were 212 Å and 111 Å for position A and position B, respectively. The vinblastine binding pocket is amphipathic (consist of both hydrophobic and hydrophilic amino acids) in nature (Table 3). The binding site residues lying within 4 Å distances of docked ansamitocin in both the positions are portrayed in Figures S2 and S3 and are listed in Table 3. An analysis of the binding site residues lying within 4 Å suggested that ansamitocin P3 binding site in both position A and position B partially overlapped with the vinblastine binding site on tubulin. The common amino acids between vinblastine binding site and ansamitocin P3 binding sites were Lys176, Tyr210, Pro222, Val177, Ser178, Asp179 of β- subunit and Lys326, Asn329, Gly354, Val353 and Asp249 of α–subunit of tubulin dimer (Table 3). In position A, ansamitocin P3 could make five hydrogen bonds with Pro175 (1.7 Å), Val177 (2.3 Å), Ser178 (1.7 Å), Asp179 (1.8 Å) and Lys 336 (2.2 Å) [32], [43] (Figure S2). In position B, ansamitocin P3 could make two hydrogen bonds with Val177 (1.8 Å) and Asn329 (2.2 Å) [32], [43] (Figure S3). Similarly, we performed docking of maytansine on tubulin dimer. The minimum energy docked conformation was found at α-β interface (Figure S4A). The estimated binding energy for this orientation is −10.26 kcal/mole. The binding site residues lying within 4 Å distances of docked maytansine are showed in (Figure S4B) and are listed in Table 3. Maytansine binding site was also found to be overlapped with vinblastine binding site (Fig. 9). The common amino acids between vinblastine binding site and maytansine binding sites were Gly354, Asn249, Val353 of α– subunit and Ser178, Val177, Tyr224, Pro222, Thr223 and Thr221 of β-subunit of tubulin dimer. In this docked conformation maytansine is making two hydrogen bonds with Asn249 (2.7 Å), and Thr223 (1.9 Å) (Figure S4B) [32], [43]. It was found that other kinds of interactions may also help ansamitocin P3 and maytansine in binding to tubulin dimer. The criteria to predict the C_α_H-O interaction, the C_α_H-O distance should be less than 3.9 Å and the angle between C_α_–H–O should be greater than 90° [44], [45]. The CH-π contacts were analyzed with a distance cut-off D_max_ 3.05 Å and angle cut-offs (angle between H-C-C’)<70° [44], [46]. For halogen (chlorine)–oxygen interaction [47], the distance between chlorine and oxygen should be less than 3.27 Å and the angle between C-Cl–O should be greater than 165° [47], [48]. In both the positions, ansamitocin P3 could also be stabilised by other weak interactions like C_α_H-O and CH-π interactions. In orientation A, Lys176, Val177 and Asn329 could provide C_α_H-O interaction. The analysis indicated that Ile332, Phe351 and Phe343 were providing CH-π contacts. In position B, no C_α_H-O interactions were found while Thr319 and Phe214 are providing CH-π contacts. Another important interaction i.e. halogen–oxygen interaction [47] was observed in position B of ansamitocin P3. In position B of ansamitocin P3, valine177 of B chain might be establishing chlorine-oxygen interaction with chlorine atom of ansamitocin P3 (Figure S3C).The presence of C_α_H–O and CH-π contacts for maytansine was also analyzed. No possibility of C_α_H–O interaction was noticed. Tyr224 was found to provide CH-π contacts.

**Table 2:** Comparison of binding properties of vinblastine obtained from molecular docking using Autodock and experiments.

		Molecular Docking	Experimental data
**Vinblastine**	Binding energy (kcal/mole)	−11.34	−14.11 [52], [53]
	Ki (at 298.15K)	4.8nM	0.16 µM [54]

**Table 3:** Residues lying within 4 Å distance of docked Compounds.

Vinblastine	Val353, Phe351, Gly354, Ile385, Asn249, Asn329, Pro352, Lys326 of α – subunit of tubulin dimer. Asp179, Pro175, Ser178, Lys176, Val177, Tyr210, Tyr 224, Pro222, Thr223, Thr221, Leu227 of β- subunit of tubulin dimer.
Ansamitocin P3:Position A	Val353, Pro325, Val328, Asn329, Ile332, Ala333, Lys336, Phe343, Phe351, Gly350, Thr349, Pro348 of α – subunit of tubulin dimer.Asp179, Pro175, Ser178, Lys176, Val177 of β- subunit of tubulin dimer.
Ansamitocin P3:Position B	Lys326, Asn329, Pro325, Ile332, Val328, Tyr357, Ile355, Tyr319, Gly354, Val353, Asp249, Leu248 of α – subunit of tubulin dimer.Thr220, Phe214, Lys176, Tyr210, Pro222, Val177, Ser178, Asp179 of β- subunit of tubulin dimer.
Maytansine	Val353, Gly354, Ile355, Asn249, Ala247, Leu248 of α – subunit of tubulin dimer.Asp101, Pro222, Ser178, Gly11, Gly225, Val177, Tyr224, Thr223), Thr221 of β- subunit of tubulin dimer.

## Discussion

In this study, we examined the mode of action of ansamitocin P3 in MCF-7 cells. Consistent with previous studies [5], [12], [49], ansamitocin P3 was found to potently inhibit proliferation of MCF-7 cells with an IC_50_ of 20±2 pM. Maytansine has been reported to have an IC_50_ of 710 pM in MCF-7 cells [10] indicating that ansamitocin P3 is more potent than its parent molecule, maytansine. Further, at picomolar concentrations, ansamitocin P3 caused severe depolymerization of interphase and mitotic microtubules in MCF-7 cells and arrested cell cycle progression at mitosis. The phosphorylation of BubR1 in ansamitocin P3 treated MCF-7 cells indicated the activation of BubR1 in these cells. Checkpoint surveillance mechanisms in the cell are known to recruit proteins that sense the detachment of microtubules from the kinetochore region. Accumulation of checkpoint proteins like Mad2 and BubR1 are known to stall the cell prior to anaphase until the defects are corrected [36] **.** Upon treatment with ansamitocin P3 an increased accumulation of checkpoint proteins Mad2 and BubR1 was observed. Ansamitocin P3 treatment activated p53 and increased the nuclear accumulation of p21 suggesting that the cells underwent apoptosis via the p53 mediated apoptotic pathway.

Ansamitocin P3 reduced the intrinsic fluorescence of tubulin and also reduced the rate and extent of cysteine modification of tubulin by DTNB indicating that it induced conformational change in tubulin. Like ansamitocin P3, several of the potent cytotoxic agents including maytansine (IC_50_, 710 pM) and cryptophycin-52 (IC_50_,11 pM) were shown to inhibit cell proliferation in picomolar range whereas these agents were found to bind to purified tubulin with dissociation constants in sub micromolar range [9], [50]. For example, cryptophycin 52 and maytansine were reported to bind to tubulin with a dissociation constant of 0.3 and 0.86 µM, respectively [10], [51]. The K_d_ of ansamitocin P3 obtained in our study is comparable with that of maytansine. Interestingly, maytansine was shown to bind to the microtubules with nearly 20-fold stronger affinity than it’s binding to soluble tubulin [10]. The binding of few molecules of cryptophycin 52 and maytansine per microtubules was found to inhibit microtubule assembly dynamics and to inhibit cell proliferation. Therefore, ansamitocin P3 may also bind to microtubule ends with much more stronger affinity than to soluble tubulin, which might explain its potent cytotoxic activity in cells.

A docking analysis indicated that the binding site of ansamitocin P3 on tubulin is partially overlapping with the vinblastine binding site. The result is consistent with the biochemical data that ansamitocin P3 competitively inhibits the binding of vinblastine to tubulin [16]–[18]. An analysis of the binding sites of vinblastine and ansamitocin P3 suggested that Phe351, Val353, Ser178, Val177 Asp179, Pro175 and Lys176 residues of tubulin are shared by both the ligands (Table 3). In position B, ansamitocin binding site completely overlapped with vinblastine binding site (Fig. 9). The common amino acids between vinblastine binding site and ansamitocin P3 binding sites for position B were Lys176, Tyr210, Pro222, Val177, Ser178, Asp179, Lys326, Asn329, Gly354, Val353 and Asp249 (Table 3). As previously mentioned, value of solvent accessible surface area is far less for position B than for the position A of ansamitocin P3, which indicated that ansamitocin P3, in position B, is more deeply buried than position A. Since difference of binding energies of ansamitocin P3 docking to tubulin at both the positions A and B were comparable, we used estimated solvent accessible surface area and intermolecular interactions to propose that position B is more preferable than position A for ansamitocin P3 docking on tubulin.

In conclusion, the results indicated that the binding site of ansamitocin P3 partially overlaps with the vinblastine binding site on tubulin and provided a new insight in understanding the binding of ansamitocin P3 to tubulin. Ansamitocin P3 potently inhibited cell proliferation by depolymerizing microtubules and induced apoptotic cell death through p53 mediated pathway.

## Supporting Information

Figure S1 Histogram of flow cytometry data of MCF-7 cells stained with only PI and Annexin V in the absence of ansamitocin P3.
(TIF)

Figure S2 (A) Docking of ansamitocin P3 on tubulin dimer in position A. C and B chains (α and β subunit of tubulin dimer respectively) are shown in red and green color respectively, and ansamitocin P3 is shown in brown. Red, blue, green and white sticks represent oxygen, nitrogen, chlorine and hydrogen atoms respectively. In this orientation, ansamitocin P3 was found at the interface of tubulin dimer. (B) Amino acids present around 4 Å of the ansamitocin P3 binding pocket in position A in tubulin dimer. Color scheme is same as in S1A. Black lines represent hydrogen bonding possibility between ansamitocin P3 and amino acids i.e. Pro175 (1.7 Å), Val177 (2.3 Å), Ser178 (1.7 Å), Asp179 (1.8 Å) and Lys 336 (2.2 Å) present around ansamitocin P3 in binding pocket.
(TIF)

Figure S3 Docking of ansamitocin P3 on tubulin dimer in position B. (A) Color scheme is same as in S1A. In this orientation, ansamitocin P3 was found to bind at the interface of tubulin dimer. (B) Amino acids present around 4 Å of the ansamitocin P3 binding pocket in position B in tubulin dimer. Color scheme is same as in S1A. Black lines represent hydrogen bonding possibility between ansamitocin P3 and amino acids i.e. Val177.B (1.8 Å) and Asn329.C (2.2 Å) present around ansamitocin P3 in binding pocket. (C) Halogen (Chlorine)–oxygen interaction for ansamitocin P3 in position B. Color scheme is same as in S1A. Chlorine atom of ansamitocin P3 is stabilizing halogen–oxygen interaction with carbonyl oxygen of Val177.B (3.24 Å). Possible halogen interactions are represented by dashed lines.
(TIF)

Figure S4 Docking of maytansine on tubulin dimer. Color scheme is same as in Figure S1A. Maytansine is shown in blue. (A) Maytansine was found to bind at the interface of tubulin dimer partially overlapping with the vinblastine binding pocket. (B) Amino acids present around 4 Å of the maytansine binding pocket in tubulin dimer. Black lines represent hydrogen bonding possibility between maytansine and amino acids i.e. Tyr224 (1.88 Å) and Asn249 (2.70 Å) present around maytansine in binding pocket. Possibility of one intra-molecular hydrogen bond was identified between oxygen and hydrogen of hydroxyl group (circled in black).
(TIF)
