# Supplementary figures and images for: Ansamitocin P3 Depolymerizes Microtubules and Induces Apoptosis by Binding to Tubulin at the Vinblastine Site
Source: PLoS One. 2013 Oct 4;8(10):e75182. doi: 10.1371/journal.pone.0075182 (PMC3790769; doi:10.1371/journal.pone.0075182)

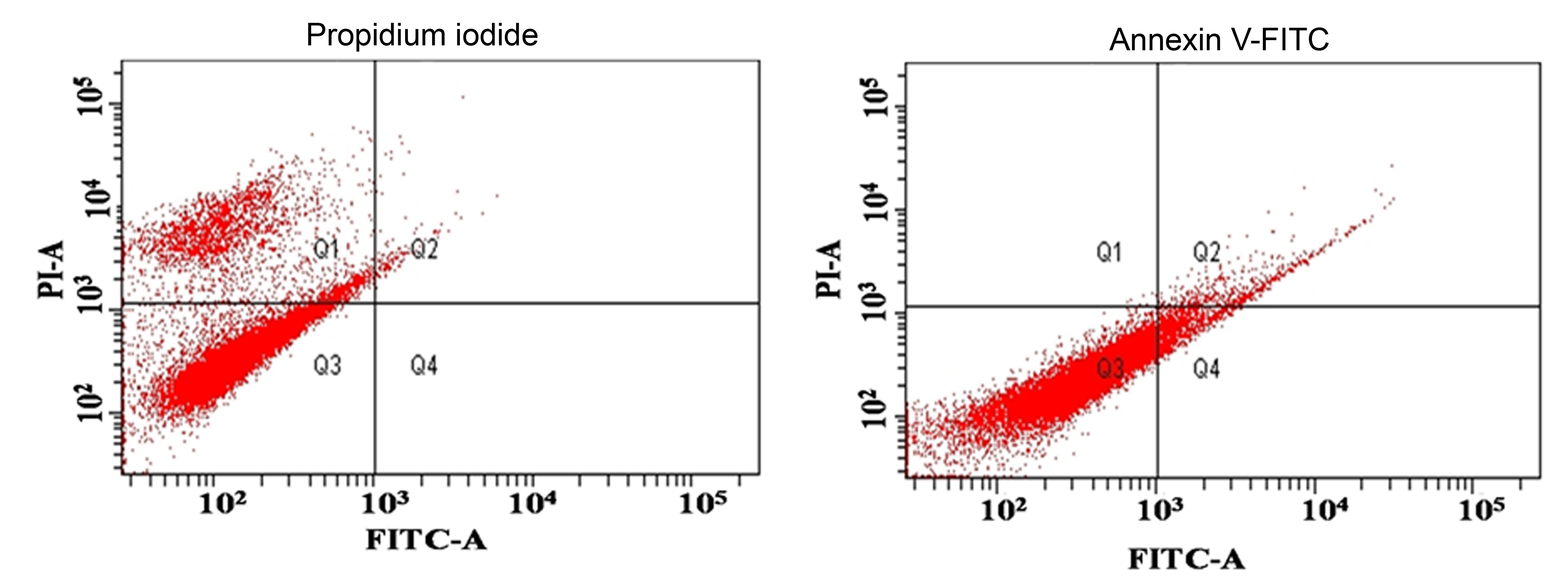

Supplement: Figure S1 — Histogram of flow cytometry data of MCF-7 cells stained with only PI and Annexin V in the absence of ansamitocin P3. (TIF) [file pone.0075182.s001.tif]

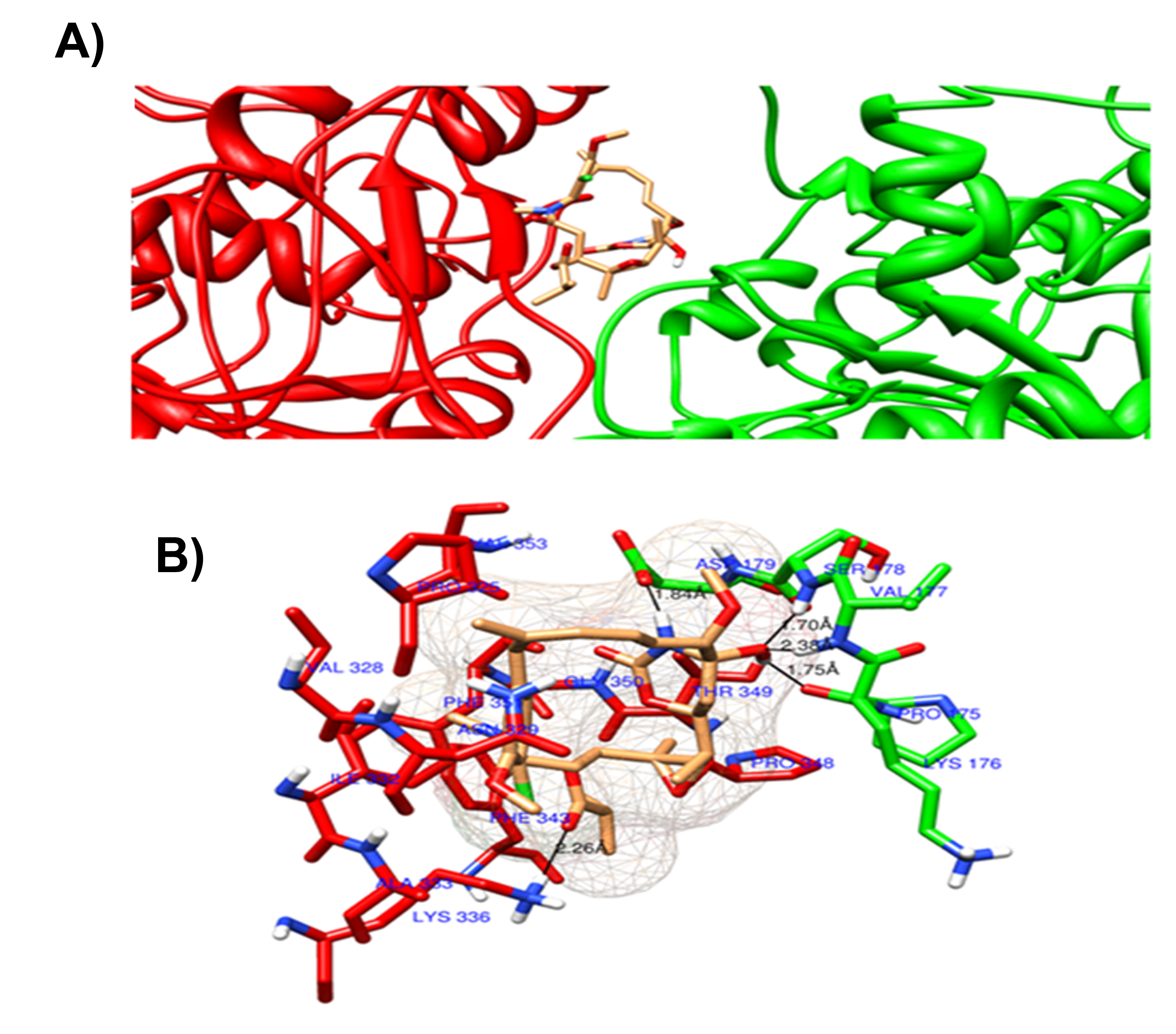

Supplement: Figure S2 — (A) Docking of ansamitocin P3 on tubulin dimer in position A. C and B chains (α and β subunit of tubulin dimer respectively) are shown in red and green color respectively, and ansamitocin P3 is shown in brown. Red, blue, green and white sticks represent oxygen, nitrogen, chlorine and hydrogen atoms respectively. In this orientation, ansamitocin P3 was found at the interface of tubulin dimer. (B) Amino acids present around 4 Å of the ansamitocin P3 binding pocket in position A in tubulin dimer. Color scheme is same as in S1A. Black lines represent hydrogen bonding possibility between ansamitocin P3 and amino acids i.e. Pro175 (1.7 Å), Val177 (2.3 Å), Ser178 (1.7 Å), Asp179 (1.8 Å) and Lys 336 (2.2 Å) present around ansamitocin P3 in binding pocket. (TIF) [file pone.0075182.s002.tif]

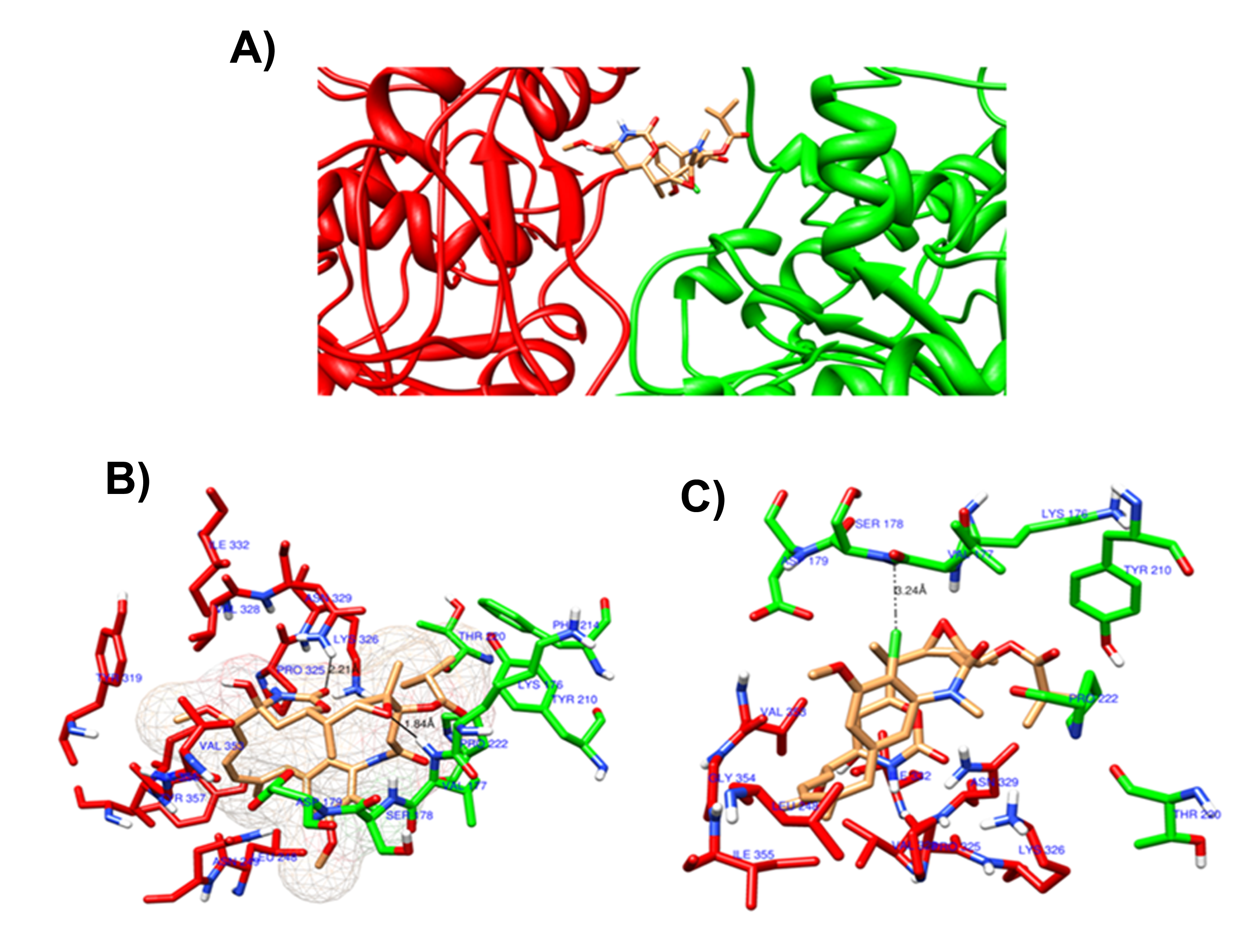

Supplement: Figure S3 — Docking of ansamitocin P3 on tubulin dimer in position B. (A) Color scheme is same as in S1A. In this orientation, ansamitocin P3 was found to bind at the interface of tubulin dimer. (B) Amino acids present around 4 Å of the ansamitocin P3 binding pocket in position B in tubulin dimer. Color scheme is same as in S1A. Black lines represent hydrogen bonding possibility between ansamitocin P3 and amino acids i.e. Val177.B (1.8 Å) and Asn329.C (2.2 Å) present around ansamitocin P3 in binding pocket. (C) Halogen (Chlorine)–oxygen interaction for ansamitocin P3 in position B. Color scheme is same as in S1A. Chlorine atom of ansamitocin P3 is stabilizing halogen–oxygen interaction with carbonyl oxygen of Val177.B (3.24 Å). Possible halogen interactions are represented by dashed lines. (TIF) [file pone.0075182.s003.tif]

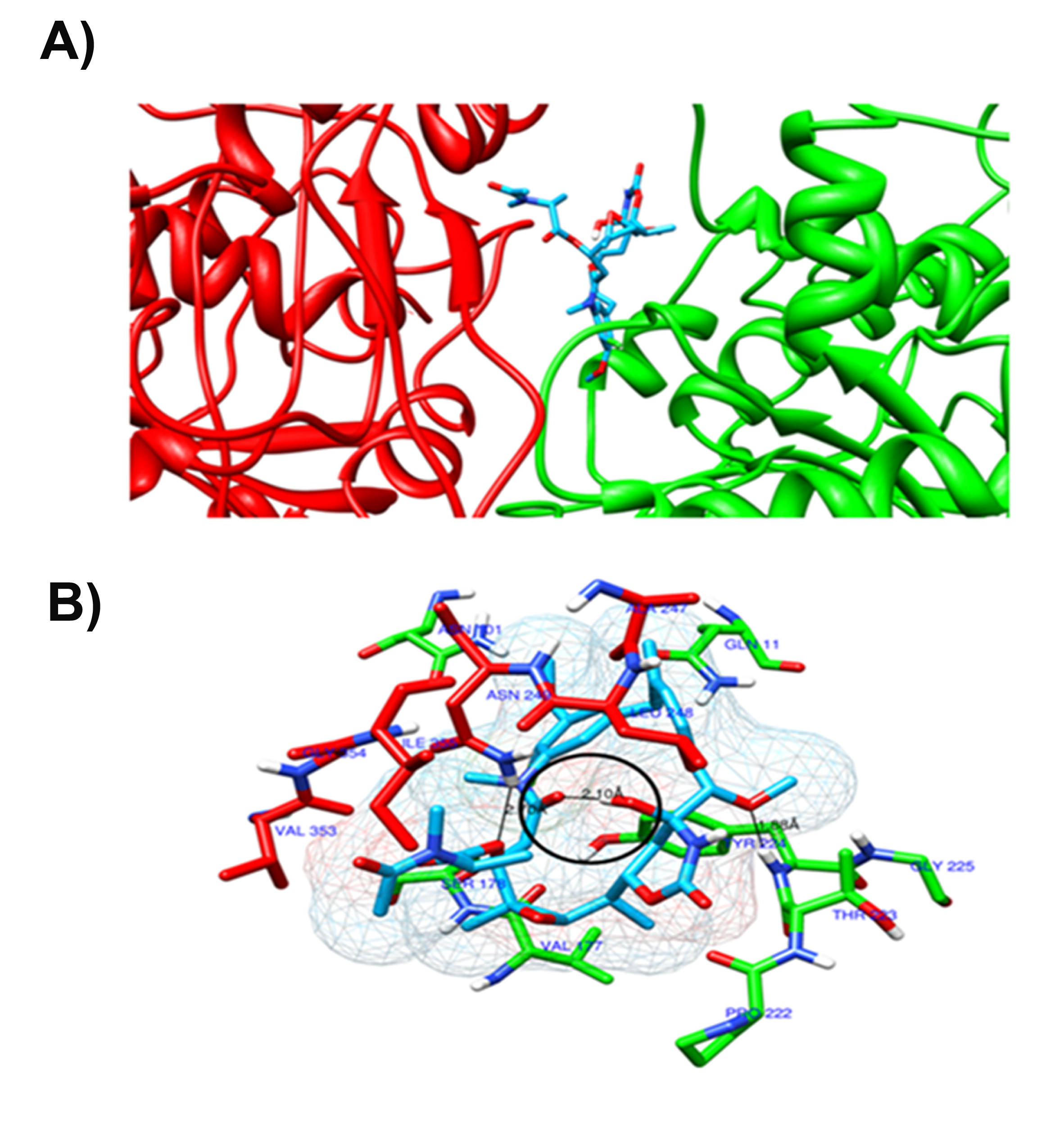

Supplement: Figure S4 — Docking of maytansine on tubulin dimer. Color scheme is same as in Figure S1A. Maytansine is shown in blue. (A) Maytansine was found to bind at the interface of tubulin dimer partially overlapping with the vinblastine binding pocket. (B) Amino acids present around 4 Å of the maytansine binding pocket in tubulin dimer. Black lines represent hydrogen bonding possibility between maytansine and amino acids i.e. Tyr224 (1.88 Å) and Asn249 (2.70 Å) present around maytansine in binding pocket. Possibility of one intra-molecular hydrogen bond was identified between oxygen and hydrogen of hydroxyl group (circled in black). (TIF) [file pone.0075182.s004.tif]
